# How to Tell a Recovery Story ‘Professionally’? Issues Related to the Transformation of Personal Stories During a Training for Becoming a Peer Support Worker

**DOI:** 10.1111/hex.70528

**Published:** 2026-01-15

**Authors:** Elena Faccio, Michele Rocelli, Giuseppe Salamina, Ludovica Aquili

**Affiliations:** ^1^ Department of Philosophy, Sociology, Education and Applied Psychology, Via Venezia 14 University of Padua Padua Italy; ^2^ Department of Mental Health, Local Health Unit of Turin Ministry Delegate for Health Turin Italy

**Keywords:** experts by experience (PSW), person‐centred care, quality service, story‐sharing of recovery

## Abstract

**Introduction:**

Sharing narratives about recovery is a central activity in peer support work. Researchers have so far investigated many issues related to recovery narratives, but have paid no attention to how the building and sharing of one's story are modified during training courses. This study examines the beliefs of participants in the first national training to become Peer Support Worker (PSW), funded by the Italian Ministry of Health in 2022–23, regarding how to craft their recovery story to make it ‘professional’.

**Methods:**

16 PSWs were interviewed at the end of the course. Implicit theories and beliefs were the focus of the semi‐structured interviews. Answers were transcribed and analysed through thematic analysis.

**Results:**

The training represents a decisive moment for socialising the ‘unwritten rules’ about how to build and tell personal experiences. Seven main themes emerged: the story‐sharing as the most important tool to understand what happened, as a ‘passage’ of status that changes the feeling between the person and the story through the presence of others, the need for a method for structuring the story, the emotion control as prerequisite for maintaining the right distance between one's story and the user's story—not imposing oneself on the other's experience, the relevance of acting as strategic story director, accepting stories without happy ending and story‐sharing for cultural and social change. Results have been discussed with the contribution of PSW, highlighting the potentials and risks.

**Conclusions:**

Training should develop competencies in expressing the story without standardising it, thereby avoiding the reification of the recovery journey into a single version. Story‐sharing should not be considered merely as the enactment of a favourable recovery journey; even stories that do not have a happy ending, or that do not adhere to the canons of the ‘recovery genre’, have dignity and social utility. In a nutshell, the PSW must be helped in becoming the filmmaker of the story rather than the performer of a static script.

**Patient or Public Involvement:**

The PSWs involved played a fundamental role in participating actively by deciding to make available their narratives, reading the results and commenting on them.

## 1 Introduction

A Peer Support Worker (PSW) is a person who, having experienced a situation of mental distress and acquired specific experiential knowledge as a service user, applies this experience and knowledge to help others [1, 2]. Stories of favourable recovery are an essential component of the helping role since their sharing may provide working tools to accompany other users in their recovery journeys, allowing them to nurture a reasonable confidence and hope for improvement [3, 4, 5, 6, 7, 8]. Numerous studies have discussed how and why listeners can benefit from hearing about the recovery experiences of others; stories have proven effective in providing users with validation, empowerment, hope and alternative conceptualisations of their suffering [4, 9].

Most PSW training courses include specific modules devoted to sharing the recovery story [10, 11], as well as enhancing introspection and the ability to describe one's experiences [2, 12, 13, 14]. Thus, how does one's story of suffering and recovery become a working tool?

### 1.1 How to Build and Perform a Recovery Narrative

Several authors have questioned how recovery narratives are built and mobilised. Woods [15] defined a recovery narrative as a ‘genre’—a particular type of story produced in specific sites. It is characterised by two aspects: ‘insight’ and ‘inspiration’. In this way, protagonists should have insight into their mental suffering; they should be encouraging and reassuring and inspire some kind of change. The credibility of the narrator, as a bearer of knowledge, comes from the successful transformation of his/her own story into a positive one (Llewellyn Beardsley et al. 2020). The recovery narrative operates as evidence, providing access to wise and balanced beliefs and reducing irrational narratives, as well as an enactment, making one's recovery real, by materialising it into a story [16]. Acceptable and unacceptable stories are thus configured. According to Voronka and Grant [17, p. 3], acceptable recovery narratives meet two requirements: to include the forms of distress that have been experienced and to emphasise ‘the individual's progress from tragedy to triumph’. Some authors have critically commented on this type of template. First, since it has been forged by a neoliberal agenda, the template carries the risk of normalising and individualising stories of hardship [17]. Second, such normativity produces mechanisms of exclusion; that is, narratives that do not fit the criteria remain unheard [16]—in particular, stories of anger and experiences of abuse, political engagement and epistemic oppression [18, 19]. Other studies have highlighted symbolic and structural constraints on personal recovery stories. Personal stories run the risk of being shaped and constrained by dominant cultural and institutional narratives and definitions given by services, which act by selecting what can or cannot be said ([3]; Sinclair et al. 2023; [20, 21]). Processes of co‐option by services in the construction of recovery stories have been observed [22, 23], such as the instrumentalisation of stories for political and institutional purposes ([24, 25, 26]; Voronka 2021). Recovery stories often draw on a model of ‘risk‐free’ and ‘positive reflection’ for mental health services, restoring professional power and roles [22]. Costa [24] offered a powerful account of coercion and compulsion in the production of a recovery narrative, highlighting the potential for disempowerment in ‘sharing one's story’ to meet the demands of mental health professionals. Finally, a risk run by recovery narrative is of being accepted and becoming ‘finished and final’, instead of being dialogic and open to flow, available to changes and revisions, meaning a risk of reification (Franck 2010). Excessive standardisation of training could limit the role that peers can offer and may result in a procedural approach [27].

Other authors have questioned the content and function of recovery narratives, as well as the directions they are supposed to follow; however, none have observed the process of making recovery narratives during a training course for becoming a PSW. Training represents a decisive moment in the socialisation of the profession's rules. It can be seen as a propitious space to follow the construction of the recovery stories. Through the analysis of the transcriptions of the interviews with 16 participants in a training course for PSW held in Italy, this study aims to investigate the process through which the experience of suffering and recovery is transformed into a professional tool at the very beginning of the path, deepening the unwritten rules they have absorbed (consciously or not) for performing the story‐sharing; that is, how a recovery story should be told in their beliefs.

### 1.2 Training Programme

The training course started in November 2022 in Borgonuovo (Bologna) in the North‐Centre of Italy. It was funded by the Italian Ministry of Health and was the first training organised at a national level, with attendees coming from all over the country. It referred to the Ex‐IN model, developed by Utschakowski in Germany (Utschakowski et al. 2008) and German‐speaking countries, Austria and Swiss Cantons. The course is divided into a theoretical part, consisting of 12 modules (each of them composed of two and a half days of residential training) and a practical part (2 apprenticeships in mental health services, lasting 120 h). The modules aimed at preparing for the sharing of personal history are those related to personal growth and the elaboration of one's life story. In particular, one of the modules is aimed at encouraging an in‐depth reflection on biography, analysing the crisis experiences lived during the recovery process. A second one addresses the theme of Self‐Determination and Relational Skills, enhancing decision‐making autonomy and interpersonal skills. Finally, the module dedicated to the awareness of resources and limits goes in the same direction, favouring a balanced self‐evaluation. All participants are invited to present to the group, at the end of the course, the reading of their recovery story. The ‘ritual’ related to the moment of sharing involves the group listening in a circle, with extreme attention, to the text of the story that has been prepared before the meeting. Listeners do not comment or enter into the contents of the narration offered. They just thank and greet the protagonist with a few words.

### 1.3 Research Aims

By adopting a narrative approach [28, 29, 30, 31], we investigated the beliefs and implicit theories related to story‐sharing as a tool used by people to organise and give meaning to their experiences. Story‐sharing means constant self‐reflection and a deeper understanding of one's experience, thanks to the possibility of giving order to previously confused aspects of one's life story [32, 33]. More in detail, the research aims to explore how participants think of turning their story of suffering and recovery into a working tool (in the peer relationship) by:

1. Investigating implicit theories and beliefs in this regard.
2. Identifying which competencies PSWs think they need to develop regarding the management of their story of ‘distress’ to play the role effectively in peer support.
3. Understanding the meanings related to the construction of experiential knowledge, starting with personal story‐sharing, and all processes that could influence story‐sharing.

## 2 Materials and Methods

The research involved 16 people, aged 30–61 years, attending the Ex‐IN programme, who had been in the course for a minimum of 10 Modules over 12 (the total). The participants in the research correspond with the participants in the course. The selection was made through CV, a letter of motivation and an evaluation interview. Some of the prerequisites for selection were: stable mental health (a criterion that involves no longer being strongly influenced by their mental health problems in everyday life, being able to deal with the problems and questions of others, and no longer being treated for acute episodes of mental distress), be able to reflect on one's own experiences and have at least one important and supportive person in life (Utschakowscki 2008). All possible histories of mental distress (psychosis, depression, anxiety and bipolar disorders) were admitted, and a criterion of territorial heterogeneity was preferred, so as to welcome candidates from diversified regions of Italy, to offer fair opportunities in the comparison between north, centre and south.

3 out of 19 participants did not complete the training course and were not interviewed because they had not achieved the requirement of sharing their story with the group. There are several reasons for early discontinuation: one person decided that neither the training nor the professional programme was suitable for their aspirations. Two other participants made a number of absences higher than allowed (22 h). The course layout gave the question of attendance as binding, and unlike what happens in Germany (where it is possible to attend similar courses for the recovery of attendance), it was not possible to make up for the training programme not attended in any other way.

The proposal to participate in the research was made through a research presentation, in agreement with the promoter of the initiative and with the two trainers, during the last training weekends. All participants freely chose to contribute and signed an informed consent form that presented the objectives and methods. They also shared the importance of publishing and disseminating the results. At the time of the interview, all participants had also completed the internship period within mental health services, so they had had their first direct professional experience in the role of PSW. A few of them were already working permanently in the services. Their socio‐demographic characteristics are shown in Table 1.

**Table 1:** Socio‐demographic characteristics of the participants.

Gender	Age at the end of the training (Years)	Participants' area of residence	Number of training modules attended
Female	61	Northern Italy	12
Female	43	Central Italy	12
Female	43	Southern Italy	12
Female	43	Southern Italy	10
Female	37	Northern Italy	12
Male	46	Central Italy	12
Female	41	Northern Italy	12
Female	59	Central Italy	12
Male	48	Northern Italy	12
Female	41	Northern Italy	12
Male	57	Central Italy	11
Female	25	Central Italy	12
Female	30	Northern Italy	12
Female	52	Central Italy	11
Male	53	Southern Italy	12
Male	33	Northern Italy	12

The trainers were also interviewed and their answers compared with those of trainers belonging to other training traditions. Since the material collected was consistent, it will flow into a second paper, soon to be published.

### 2.1 The Trainers

There were two ex‐in trainers: the first was an expert by experience, and the second was a sociologist and expert in the history and practice of intervention in the mental health system. Their Ex‐IN training for trainers had been carried out in German, a few months before the start of the Italian training. The two trainers maintained a constant supervisory relationship with their training agency, the Ex‐In German Network, in order to share all the choices regarding the training and monitor the progress of the experience, since it was their first course in the role of trainers.

### 2.2 The Interview

The questions that made up the semi‐structured interview were conceived based on the Ex‐IN training objectives and on the main issues available in the literature on the evaluation of the effectiveness of the programme to become PSW. The themes investigated, along with related questions, are presented in Table 2.

**Table 2:** Aims and questions composing the semi‐structured interview.

Investigated area: Personal experience with the course	Questions composing the semi‐structured interview
1 Difficulties and perception of the context/climate for sharing personal story 2 Perceived risks and benefits in telling about oneself in such depth 3 More or less appropriate formulas for telling one's story 4 Consequences and implications	1.1 Did you have any difficulties in sharing your personal story during the course? 1.2. What helped you do it? 2.1 Do you think that telling your personal story involves risks or/and benefits? 2.2. Which ones and how to prevent them? 3.1 How did you construct and tell your story? 3.2. Did you change your belief about how to construct your story during the training? 4.1 What did it mean for you to tell your story during the training? What effects did this have?

In order to adapt the language to the way of speaking of PSW, the construction of the questions was also helped thanks to a focus group (or pre‐research) that was held during the last weekend of the training course (May 2023).

The Ethics Committee of the School of Psychology of the University of Padua approved the research project (protocol no. 4862 of date 30/06/2022). The consent form was shown to each participant beforehand, and any possible doubts or questions were discussed.

### 2.3 Data Collection

Following a qualitative research approach, the data were collected through individual interviews, which allowed deeper and more personal discussions with each participant.

These interviews were conducted in 3 months, from April to June 2023. The data collection and analysis are based on the guidelines of the qualitative research [34]. We preferred the semi‐structured narrative interviews because they allowed us to focus on the meanings and the topics we were interested in, for a better understanding of other significant elements that could emerge during a conversation, rather than a more schematic test. The interview, in fact, is considered not only a tool but also a method, because of the interaction process established between the interlocutors. We realised one‐to‐one interviews in the context where the course was held (Borgonuovo, Bologna).

The interviews were conducted by E.F., L.A. and M.R., who also participated in the analysis of the narratives that emerged. From a narrative perspective, the interview is not conceived as a mere data collection tool, but as a space of discursive co‐construction, in which meanings and understandings are shaped through the interaction between the narrator and the listener (Mishler 1986 in Riessman 2008). In this context, it becomes essential to make explicit our positioning as researchers and to reflect on how our identities, experiences and roles influenced the process of data collection and interpretation. Researchers introduced themselves to participants as psychologists/psychotherapists, as well as researchers, experts in qualitative research methodologies, and specifically trained in the analysis of autobiographical stories. They were aware that this dual identity would shape relational dynamics. Prior to data collection, they dedicated time to familiarising themselves with the group, building relationships that made the encounters more natural and less formal. During the interviews, participants were free to choose which of the three researchers they wished to speak with, a choice likely influenced by factors such as gender, age or common regional background, although these factors were not always explicitly stated. Interviewers were careful to acknowledge that asking participants to narrate their personal stories could evoke emotional vulnerability. Participants were also given the freedom to choose the most appropriate moment to conduct the interview. Interviews were held in private and calm spaces, with the aim of fostering a climate of safety and mutual respect. The interviewers' psychological training oriented attention towards subjective meanings, encouraging narratives that connected personal biography with the training experience, rather than with diagnostic or psychiatric information. Participants were aware that researchers recognised and valued the PSW as playing a central role both within and outside mental health services: the fact that their contribution was considered competent helped reduce the typical asymmetry of the interview situation. As Riessman notes (2008), although power relations are never equal, disparities can be mitigated. In our view, this positioning likely facilitated the emergence of more open, reflective and symmetrical narratives, in which the expertise of the PSW was actively valued.

### 2.4 Data Analysis

The collected interviews were audio‐recorded and transcribed, and then analysed, according to a constructionist point of view.

The responses provided during the interviews were transcribed verbatim and analysed by the three researchers (E.F., L.A. and M.R.). The thematic analysis process was conducted following the guidelines of Braun and Clarke, which consist of six distinct phases: familiarisation with the data, generating initial codes, searching for themes, reviewing themes, defining and naming themes, and producing the report. Immersion in the data began during the conduct and transcription of the interviews. As previously noted, the authors who conducted the interviews also participated in their analysis, establishing a direct link between the interpretations emerging during the encounters and the analytic phase. The process of reflexivity is fundamental in qualitative research. During data collection and analysis, all authors kept brief reflective notes and meeting minutes to record hypotheses, emotional reactions and analytical decisions. These materials informed discussions on coding and the refinement of themes. These notes were not analysed as data; rather, they were used to document an audit trail (from excerpts to codes to decisions) and to check against premature closure. Subsequent thorough rereadings of the transcripts facilitated the approach to coding, allowing the identification of the most significant segments in relation to the research objectives. Coding was performed manually, assigning concise labels to each excerpt that represented its content. After coding, the three researchers discussed their individual codes and worked collaboratively to construct the themes, ensuring agreement on the interpretation and grouping of codes. The resulting codes were then grouped according to shared meanings or common concepts, leading to a first overall version of the themes, which was subsequently reviewed. During this phase, the internal coherence of the codes with respect to the themes and the ability of each theme to reflect the meanings present in the data were verified, resulting in the merging, splitting or elimination of some initial themes and the production of a more coherent and refined version. Finally, after careful rereading of the excerpts, a comprehensive report of the data was produced, selecting the most illustrative examples to represent the emerging themes, which are presented as the results in this article. For clarity and to guide the reader, Table 3 presents the codes associated with each theme.

**Table 3:** List of the seven themes and their respective codes.

Theme	Codes
Telling others for a better understanding of one's recovery journey—storytelling as a ‘passage’ of status	– Telling the story to the group as a process of emancipation from suffering – Resignification of one's story through sharing
Experience of telling is not enough; you need a method to tell your story	– Training as a narrative structure for the personal story – Knowing how to share one's story demonstrates one's recovery
Controlling emotions related to one's suffering	– Constructing the story to safeguard emotions – Selecting what to share in the group
Keeping distance between one's own story and the user's story—not imposing oneself on the other's experience	– ‘Professional distance’ to protect oneself – ‘Professional distance’ to protect the other
To act as the strategic director of your story	– Reformulating the story according to the audience – Using the positive story for the benefit of the other
Stories without happy endings	– Patience in not fully understanding the other's story – The positive story does not always support the other
Storytelling for cultural and social change	– Telling one's story as a collective act – Narration as a tool for destigmatisation

We shared the results with the participants in two final sessions, during which we not only introduced the results but also asked for feedback and thoughts about what their most important result was, and we are reporting on this in this article. These steps increased transparency without treating the reflexive artefacts as part of the empirical corpus.

## 3 Results

### 3.1 First Theme

#### 3.1.1 Telling Others for a Better Understanding of One's Recovery Journey—Story‐Sharing as a ‘Passage’ of Status

The training course provides a space for participants to share stories of suffering within the group. This activity was considered very demanding from an emotional point of view; it was difficult to prepare and face the moment of reckoning or reading the written story, but the welcoming and participatory atmosphere was fundamental for everyone. Telling one's recovery story to the group was described by many as cathartic, an immersion in memories and awareness. The self‐revelation becomes part of the recovery process itself.

The passage was when they told us “by such day you have to write your recovery story and you have to talk to the group for 10 min about it” […] Then I wrote it and many things came out that I didn't expect to feel, that is, I managed to free myself completely and maybe it was what unlocked me in all respects, because of the first recovery (editor's note: the first story of the recovery) you tell all the bad things that I didn't even dare to remember. That was just a moment when I had to unravel the whole truth, and somehow I assimilated it.

P12

In my opinion, sharing lightens, sharing is part of the cure, of therapy. […]

P2

The term ‘therapy’ refers to the co‐participation of others by talking about themselves. It is a process of emancipation from suffering in solitude that is realised through disclosure. Telling others means changing the relationship between oneself and one's story, not being confined within a private dimension anymore.

[…] one tells one's story more than anything else for therapeutic reasons; there may be points in one's story that perhaps one has not solved, by sharing it perhaps you can dissolve those points that you have not solved … many times, yes, it has therapeutic functions.

P5

The ability to change point of view, to change perspective, is closely linked to the group experience. The comments offered by others, precisely because they are considered competent concerning the experiences of suffering, allow stories to be re‐signified.

I also began to lighten up a bit and emphasise the dramatic aspects of my story less. That is, I felt I had managed to repair; there was a repair of … a light that I gave … a choice to come out into the open, to come out.

P15

An active participation of the audience, the possibility of receiving feedback and comments, not only facilitates expository practice but also assigns new functions to it, such as that of instilling a sense of cohesion, unity and identity to the group, which, in turn, encourages further sharing of experience.

Well, certainly others are more objective, see things that you can't see. There were comments that a person wouldn't think about, and so, others gave you the possibility to have another point of view. If I saw certain things as negative, other people made me realise that they weren't so negative.

P11

The group entered powerfully into another person's story, providing unforeseen suggestions not contemplated by the perspective or situated gaze of the narrator. Story‐sharing is connected to a process of reflexivity that allows one to process one's past and respect that of others.

I respect my story. I don't need to throw it up to you to hide a request for help.… I gained the ability to express myself consciously.

P15

Reflexivity allows one to distance oneself from one's lived experience, recognise one's limits and value the transformative meaning of the story. It helps not only to tell but also to ‘be’ in the stories of others without overlapping or blurring.

Well, in the meantime, the group's journey and group work have led us to reflect on many things, also to confront each other in a very deep way on many issues. So on the meaning of the diagnosis, for example, it was a theme that struck me a lot, because for me it was not a problem, for others, they experienced it with suffering. So to understand, in short, and also to give value to things that I had never considered so important, and instead thanks to the group I thought: I can also share it.

P13

Telling one's stories and listening to those of others allows one to relocate many issues, to resize the seriousness of the events and to confront the meaning of the diagnosis, with the theme of passivity or activity concerning the construct of mental illness.

### 3.2 Second Theme

#### 3.2.1 Experience of Sharing Is Not Enough, You Need a METHOD to Tell Your Story

The first requirement to be credible as a PSW is to be able to demonstrate an adequate recovery path. The course offers tools that the HPSW must be able to put into practice.

I think it's very important to have a good recovery and a good training. […] If you tell me that you have awareness, they have given you the tools but when you are sick you cannot use them, you do not have a good recovery, because if someone gives you tools, you must know how to use them.

P13

Unlike people who have only experience but no training,… I know I have a basis on which I can act to help a person.

P10

The training provides the structure and the basis of the working competence. Once one's experience is placed within a narrative system, the story itself becomes an element capable of certifying the successful elaboration of one's recovery story. In other words, one's competence in story‐sharing becomes proof of one's recovery:

Until you can tell your story, you haven't elaborated on it. Why do some people struggle to tell it? Because they don't yet have such a clear idea.

P14

Story‐sharing is proof of healing; it not only tells it, but realises it. The ability to narrate becomes the litmus test for one's recovery. Self‐understanding is conceived as a preliminary step, necessary but insufficient, in transforming one's experiences into a working tool. Guidelines and a method are needed.

When you work, you need a method to share the recovery story in the right way because someone might also feel very touched, and so it takes … an ability to learn how to share one's experience, and the course gave me that.

P11

[The course] helped us be more structured in talking about our experiences, our stories, our lived experiences in facing various situations, also towards others.

P2

Terms such as ‘method’, ‘learning’ and ‘structured’ highlight how the process of professionalisation requires the narrative of one's experience to be adapted within specific boundaries. A normative dimension of the story emerges, capable of making it legitimate.

Training is needed to have a method, to learn … strategies to know how to relate, to know how to manage one's emotions. If you don't know how to do these things here, to be an Esp … you can also do damage eh…

P15

The ‘method’ concerns first of all the ability to manage emotional aspects, as we will see better in the next theme.

### 3.3 Third Theme

#### 3.3.1 Controlling Emotions Related to One's Suffering

During the Ex‐IN training, stories of suffering and recovery were progressively smoothed out and rehashed. As can be seen from the excerpts below, not everything one had experienced was shared with the group.

Certainly, with the course, the way I tell my story has changed. There are parts that I removed because they were too intimate, stories too strong to be shared with the group. Even if others dared to share certain situations, I didn't have the courage. In any case, mine is a linear narrative; there are no major heavy events, and mine has been a soft recovery.

P10

Having been the first of the whole group to tell the story, I didn't really tell when I was sick. Let's say that I focused more on the solution than on the problem. So I didn't go looking for evil so much; I told a little bit about how I got out of hell in my own way.

P14

When sharing the story with the group, some parts are deepened, while others are cut out. Episodes that are considered too intimate and deep or too strong to tell are kept private. The central issue is not the blurring of certain parts, but rather the reasons behind this choice. For instance, the idea of ‘not having the courage’ may highlight the risk of the establishment of guilt processes towards a story that is perceived as ‘incomplete’, intentionally partial, not authentic, or not true [35].

Well, the risk is that if one hasn't worked well on sharing the story—it's very tough, in my opinion—you will only tell bits and pieces … or you will start to re‐move things inside, and there—I think that, emotionally—I felt a bit shaken at times, but I had my support points. Other people may risk re‐opening the wound, even marginally.

P6

Working on one's own story, sharing it with others and measuring oneself against the effects that the story produces in oneself becomes the elective strategy for managing those emotions and not being overwhelmed by them. It is therefore necessary to work on emotional control.

Look, the risk is that confronting the other person's discomfort can generate mistrust, and mistrust leads a bit to sadness, to depressing yourself. The risk can be a loss of trust—in short, to be overwhelmed by situations that sometimes we don't want to talk about but are extremely problematic in terms of suffering and, therefore, sometimes to be overwhelmed by life.

P9

I try not to get too involved, in the sense that I put up stakes.… But this is also what the course taught me: that you need stakes and that I have to defend myself; that is, if I go into crisis, which is possible,… I will hurt myself, so I have to protect myself.

P13

The course taught how to control overwhelming feelings and how to handle comparisons or identifications between participants, avoiding the onset of a crisis. Thus, understanding the limits of what can be told or said becomes a necessary step in the process of professionalisation in the role of a PSW.

The real recovery is when you manage to talk about your story without “Oh my God!”. If I go back and think about it for a moment, I'll feel the same emotions that made me enter the crisis.

P14

Interestingly, the story creates emotions that could resemble those felt in its time, or creates the complicity of those who know what happened, but do not let themselves be overwhelmed, as if they were talking about someone else and not about themselves.

### 3.4 Fourth Theme

#### 3.4.1 Keeping Distance Between One's Own Story and the User's Story—Not Imposing Oneself on the Other's Experience

Although the first‐person experience represents a shared element that allows participants to recognise themselves as PSWs, the need to protect the uniqueness of one's and others' stories strongly emerges. Preserving this uniqueness while enhancing the differences is not only an act of mutual respect, but it also takes on an ethical value of safeguarding the intrinsic and unrepeatable value of each story.

One's experiential knowledge has limits, and it should not be confused with the person's experience. It is complicated to use experiential knowledge because it is easy to fall into a trap. That is, is it my experience that is told, or is it the experience of the person I am accompanying that is told? It takes a lot of work.

P8

When you talk about your experience, you talk only about your experience. This is my experience; it is not necessarily yours. I have drawn this from my own experience. I can give you a cue, but then it's up to you to recognise the cue and throw it out or not. The experience is unique. Even if there are logics and regularities, the experience remains individual.

P9

The construction and protection of one's professionalism is achieved by drawing on structured and recognisable elements. For example, the exercise of ‘professional distance’ as a theme of conduct that regulates interaction protectively and recognisably.

One can enter into too‐close intimacy, so, indeed, a PSW, with empathy, with listening and with a series of skills and human qualities can get very close to the other person, but there is always that minimum of “professional distance”—let's call it like that—that must be maintained, and entering into resonance could mean perhaps putting oneself at risk and putting the work of the service at risk.

P1

I had to learn to keep my distance a bit. The educators pointed out to me that I was too involved. You have to find the right distance. How can you not get emotionally involved with a person who is suffering, especially if he is your age?

P12

### 3.5 Fifth Theme

#### 3.5.1 To Act as the Strategic Director of Your Story

One learns to tell stories, that is, one learns to renew them, each time, on the basis of reading the context, which allows one to select what is most useful to say or not to say.

I have mainly given testimony in schools; there, you must be very careful in what you say because, especially if you talk about self‐harm or suicide or if you talk too badly about the mental health service, you can negatively influence people listening to you, and so many people are sick! So, you have to pay attention not to scare people, and young people. I always try to give a positive testimony. I also choose what to say about whom I have in front of me.

P12

Awareness of context and the ability to adapt one's narrative are hallmarks of a transformative storyteller. Story‐sharing becomes a bridge between one's personal experience and the collective need for awareness raising by avoiding falling into stereotypical or overly dramatic narratives.

The testimonies are not all the same […] Every time you say what you feel like saying. We introduce ourselves as facilitators, we explain that we have made a recovery path like this … I more or less always say this. But then after … each testimony told something different, what seems most suitable to me.

P12

The ability to act as directors of one's story, without considering it a mere script to be recited, but managing to choose which parts to work out and which to leave behind, appears to be an indispensable component. There is an awareness that there is no single truth of a story, given once and for all. The story is co‐built every time with those in front of you, selecting, choosing, with empathy and attention to the needs of those to whom the story is told.

It is interesting to note the appearance of the ‘we’ to testify to the passage from the ‘I’ as a person to ‘we’ as HPSW. Professionalisation is evidenced by the use of pronouns.

### 3.6 Sixth Theme

#### 3.6.1 Stories Without Happy Endings

Telling about oneself also means understanding one's needs better and earlier. The story should not please the expectations of the other, nor must it entertain him.

[…] In the past, I had the problem of pleasing, of not being able to say no, I was entangled […] Now, if I want to say my thoughts, my experiences, I don't need to be crazy or to crack. I have acquired the possibility of being able to express myself […] I should not enchant you, I should not entertain the other. Finally, I see myself and finally I see the other […] In the past I had this communication difficulty, my inner […] Now I respect my story, I don't need to vomit it to hide a request for help'.

P15

The conclusion is masterful that not everything can be understood. Human affairs escape any control. Their mystery is expressed here, which not even stories can exhaust. Human complexity must therefore be ‘contemplated’ in its richness. It requires patience and respect; it does not always have solutions.

With the knowledge of other stories, you understand that there are dynamics that will never be controlled and for which you have to have respect and also bring a sort of “not impatience” […] therefore sometimes we must also be contemplators of what is happening. […] the course helped me to understand the other perspectives and to have patience in that sense […]

P2

### 3.7 Seventh Theme

#### 3.7.1 Story‐Sharing for Cultural and Social Change

Story‐sharing is conceived by the participants not only as a transformative tool of individual history, but also as a driver of social and cultural change. It becomes a collective act capable of changing perceptions, breaking down stereotypes, deconstructing stigma, both internal and external, and redefining identities.

The training made me make this change; it made me accept everything that had happened.… Before, I was ashamed. I felt like a thief because, yes, it's true that you don't choose your illness. It's just that when someone has a bad illness, for example, he/she is someone who has a bad illness—but in the case of mental illness, you “are” the mental illness; that is, I carried this stigma.… I became my diagnosis. By sharing my story, I realised, by writing it down and then sharing it, that it wasn't my fault, that I didn't have to be ashamed, and that I could be of help to someone else who was ashamed like me.

P12

Conscious story‐sharing is not only an act of expression, but also a practice of identity re‐appropriation, in which the storyteller is no longer defined by his or her experience of suffering, but he/she becomes the bearer of a message of possibility.

## 4 Discussion

This study aimed to investigate the process through which one's experience of suffering and recovery is transformed into a professional tool. The results align with research indicating that during training, the process of professionalisation into the PSW role proceeds hand in hand with a movement of structuring and systematising one's history of suffering and recovery [2, 7]. As acknowledged by Hegedus et al. [13], the training provides, albeit tacitly, guidelines to inform participants about the kind of story that can be told. The ability to place one's memories within a narrative structure allows participants to observe themselves as professionals. In other words, by giving their stories a degree of control, participants begin to shape their identity as PSWs. But what are the steps through which this redefinition of one's history and, subsequently, the role change take place? The interviews reveal that the transition occurs in two steps: first, perceiving oneself as skilled in self‐sharing, and second, perceiving oneself as capable of controlling overwhelming emotional responses. Both are considered elements that attest to the achievement of recovery. Consequently, one's experience is transformed into a ‘recovery story’, making it conceivable as a working tool [36].

Our research, unique in the literature concerning this objective, identified a series of elements that participants associate with the production of good recovery stories and the good storyteller. A sort of ‘implicit theory’ on the ‘story‐sharing method’ emerged. The sharing of the story is understood as a passage of status for entry into the PSW's role; it helps to understand one's suffering, to show it and to ensure that others may benefit from, ‘fully implementing’ the recovery path.

The story must have a structure, a method; it must be able to maintain the distance; it must respect otherness; the narrator must know how to control the emotions related to one's and others' suffering; it must be composed according to a positive ending, be a source of inspiration and validation of their experience by the other members of the group, and must adapt to contexts and interlocutors; and should not destabilise them if they are in a phase of fragility. It is experienced as an opportunity for reflection, increases self‐awareness and self‐monitoring, and takes on a therapeutic function. It can train the PSW to become aware that not everything can be understood or rationalised.

It seems to us that these results offer favourable solutions to the three main issues that animate the debate of the literature on the subject, which we summarise below: (1) How to guide in shaping and expressing one's recovery story without standardising it [37, 38] and turning it into a ‘procedural’ approach to the role [27]. In sharing these stories, there is a risk that individual narratives may be reduced to static representations of a generalisable and knowable collective body (Aquili 2024; [27, 39]). In line with [36] reflections, this leads to questions about how the narrative structure proposed by a training can foster solid identity construction while avoiding imposing standards that limit personal expression.

Our 16 interviews allowed us to grasp how important it is for the PSW to have a ‘structure’, a ‘method’ that makes them feel more competent than a simple user. Results have shown that the solidity of the ‘method’ is strongly associated with the solidity of professional competence. It guarantees legitimacy and protection, particularly at the beginning of the path. Perhaps the critical point is therefore not to have a ‘structure’ to compose one's story, but to what extent this should be interpreted rigidly or flexibly. It seems to us that the proposed training did not dictate too stringently the canons for the construction of the story, but offered strategies to control emotions, manage distances and not identify with the story of the other: valuable and necessary skills.

A second thorny issue in the literature concerns the emphasis placed on ‘THE story of one's recovery’, as if it were singular, one and only one, and the one shared during the course. Could the training crush one's past by channelling it exclusively into a story, perhaps the one related to the path of mental illness? Based on the findings, the reification of the recovery journey into only one version of the story, always making it the same in a static form and defined according to a narrative canon, is avoidable. Recovery can be considered as a fluid and ever‐changing journey [40], and the risks of crystallising it into a finite narrative, which becomes the main criterion for validating a PSW's professional identity, may be avoided (Franck 2010).

A third dilemma concerns the relationship between story‐sharing and recovery. The ability to tell one's story effectively is often interpreted as an indicator of the achievement of recovery itself. This implies that the course not only trains PSWs but also seems to suggest a kind of normativity about good and bad recovery narratives, in which story‐sharing skills become a metric of recovery. The thematic analysis also highlighted this link between good recovery and the acquisition of the strategies proposed during the course, between favourable recovery and the ability to tell it.

The narrative is charged with symbolic meaning: if you can't tell your recovery story, you feel guilty because you haven't recovered—so the inability to tell becomes another experience concerning which you have failed [35] cited in [19]. Thinking specifically about our participants, we also have three missed stories, those of people who have not completed the course. One wonders if they are lost forever? ‘Where do the stories of those who have not completed the path to become PSW, of those who have not been successful, the cyclical experiences of relapse, which have no happy ending, of psychiatric abandonment, end?’ (Wood 2021). This third point deserves a lot of attention; our participants seem to be aware of the fact that even stories without happy endings must find dignity and space. Their account can be even more useful than the canonical stories, because they testify that not everything is comprehensible. Stories of abandonment or relapse can be more authentic and effective, and perhaps future research should investigate the power of these kinds of stories rather than keep them away.

These three dilemmas can be made to evolve and can become generative of new ideas where the course trainers set themselves the goal not only of having the PSWs tell their stories, but also of favouring the development of their agency, in the role of narrator. The results of our research seem to confirm that courses can be oriented towards training in more or less passive ways, as storytellers or as directors of their history. The storyteller tells the story on command as a theatrical performance, activates the script of the story, and in doing so, ‘performs’ his script. The director of a story cuts pieces of it according to the moment, the needs expressed by the scene, and according to the director's objectives, which can change from moment to moment. The PSW should not be a victim of his role and suffer the obligation to tell. To enhance himself and his story, without having to ‘spill the beans’, he/she can enter the game of stories and become a strategist who chooses what, how and when to tell, but also when not to tell at all. Not everything must be told at all costs. Just as it is not true that not sharing parts of oneself that disturb does not mean pretending or denying or not having accepted them. The relationship with stories can be creative and plural, in the name of possibilities. We believe that training on this, on the story as a plausible creation, and not as the only proof of the truth of events, can be fundamental in the delicate moment of transition to the role [39, 41].

## 5 Conclusions

It should not be underestimated how much the course setting may ‘forge’ the stories of recovery. Indeed, narrative training runs the risk of compressing an individual's past within a predefined schema, transforming a personal experience into a generalisable and recognisable element [8, 21]. It is important that there is an awareness of this on the part of both trainers and PSWs, and some of the results of our research could become an integral part of the training modules aimed at talking about the topic. Training courses should increase the management of complexity and offer the PSW the role not so much of those who recite a script that is always the same, but of an active director of their story, orienting them towards reflective, strategic and contextual story‐sharing, able to choose what to say and what not to say based on competence concerning their emotional condition and in full awareness of that of the user or listeners. The PSW will thus choose to tune in with the other based on precise objectives, shaping and reshaping it creatively [42].

### 5.1 Clinical Implications

The power of stories lies in their generativity and transformability [43, 44]. The change in identity can, in fact, be identified by how people transform the story of themselves. Building new plots is, however, a journey that never reaches its destination. For the PSW not to saturate the story by exhausting it all in the narrative of recovery, not to empty his/her identity through professional experience, remaining devoid of private stories to manage in the intimate world of personal affections, they must be helped to respect the sacredness and plurality of stories. A risk of the recovery narrative is that it is accepted and becomes ‘finished and definitive’ instead of being dialogical, open to flow, change and revision ([45]; Franck 2010).

In work based on lived experience, not everything needs to be shared. That which remains unspoken, private or not yet articulable constitutes a professional resource rather than a limitation. Preserving such areas of opacity helps maintain the multiplicity of the self and protects the continuity of personal identity, preventing professional experience from overwhelming or consuming the intimate dimension. Training programmes and supervision sessions should therefore promote not only the right to speak but also the right to remain silent: a reflective space to consider what is appropriate to withhold and how to safeguard the boundaries between personal life and professional role.

Once shared, stories tend to circulate within work contexts, being cited, referenced or repurposed. Without careful attention, they risk becoming institutionalised narratives, losing their generative potential. Introducing moments of ‘collective maintenance’, in which work groups reflect not on the content itself but on how narratives are used—who tells them, who reuses them, in which contexts and with what effects—helps preserve the dialogic and plural nature of experiences, preventing them from becoming mere rhetorical tools or fixed professional identities [46, 47, 48].

A final clinical consideration concerns the relationship between this type of group experience and psychotherapy [49]. Some references to psychotherapy emerged: during role‐playing activities and the sharing of recovery stories, some participants felt that more personalised psychotherapeutic support was lacking. The presence of such a figure would have been useful in managing the intense emotional reactions aroused by reliving past experiences and would have allowed participants to take a more reflective stance towards themselves. This would have created a space for self‐assessment of their ability to emotionally cope with the most critical experiences [50]. Furthermore, it is conceivable that during the story‐sharing activities, relational processes similar to those that occur in mutual self‐help experiences were triggered: the sense of closeness and empathetic solidarity among participants seems to have contributed greatly to the action of support, reinterpretation of one's own story and displacement/rescaling of experiences, somewhat reminiscent of the experience of ‘clinical work on experiences’ typical of group therapy.

To some extent, the group may have taken on meanings related to the therapeutic experience. However, the comparison with psychotherapy is difficult to investigate and describe. It can be imagined that in group therapy, the intervention is more structured and professionally guided, capable of addressing even more complex psychological problems, but the analysis of connections, overlaps and differences remains to be explored in greater depth.

### 5.2 Limitations

The study focused on a limited number of participants within a single training course, which may affect the generalisability of the findings. It was also the first course managed at the national level and financed by the Ministry of Health; therefore, this type of training remained free from the logic of local services and allowed the involvement of PSWs from all over the country, in a very heterogeneous way. This may have favoured the independence of the course and the trainers, making their contribution very aseptic concerning the logic of local power. We believe that this can represent an added value, but at the same time, it may have made the results unrepresentative of other courses. Future research could compare this type of training with that offered in other traditions.

## Author Contributions

**Elena Faccio:** conceptualisation, data curation, formal analysis, funding acquisition, methodology, project administration, writing – original draft, writing – review and editing. **Ludovica Aquili:** data curation, formal analysis, investigation, methodology, writing ‐ original draft, validation, writing – review and editing. **Michele Roceli:** data curation, formal analysis, investigation, methodology, writing – original draft, validation, writing – review and editing. **Giuseppe Salamina:** funding acquisition, project administration, supervision.

## Ethics Statement

Approval for the study was obtained from the Ethical Committee of the School of Padova, at the University of Padova (approval 4862). All patients provided written informed consent prior to enrolment in the study.

## Consent

The participants were informed of their right to withdraw at any time. They signed a written informed consent form regarding their participation in the research and its publication.

## Conflicts of Interest

The authors declare no conflicts of interest.

## Academic Proofreading

The paper has been linguistically proofread by the Scribendi online service.

## Data Availability

The data that support the findings of this study are available on request from the corresponding author. The data are not publicly available due to privacy or ethical restrictions.
